# [4-*tert*-Butyl-2,6-bis­(di­phenyl­meth­yl)phenolato-κ*O*]dieth­yl(tetra­hydro­furan-κ*O*)aluminium

**DOI:** 10.1107/S2056989018001172

**Published:** 2018-01-26

**Authors:** Mikhail E. Minyaev, Ilya E. Nifant’ev, Andrei V. Churakov, Andrei V. Shlyahtin

**Affiliations:** a A.V.Topchiev Institute of Petrochemical Synthesis, Russian Academy of Sciences, 29 Leninsky prospect, 119991, Moscow, Russian Federation; b Chemistry Department, M.V. Lomonosov Moscow State University, 1 Leninskie Gory Str., Building 3, Moscow 119991, Russian Federation; c N.S. Kurnakov Institute of General and Inorganic Chemistry, Russian Academy of Sciences, 31 Leninsky Prospect, Moscow 119991, Russian Federation

**Keywords:** crystal structure, aluminium, phenoxide complex, 4-*tert*-butyl-2,6-bis­(di­phenyl­meth­yl)phenol, caprolactone polymerization, NMR

## Abstract

The title compound, {Al[O-2,6-(Ph_2_CH)_2_-4-^*t*^BuC_6_H_2_]Et_2_(THF)} or [Al(C_2_H_5_)_2_(C_36_H_33_O)(C_4_H_8_O)], was formed in the reaction between 4-*tert*-butyl-2,6-bis­(di­phenyl­meth­yl)phenol and tri­ethyl­aluminum in the presence of THF (THF is tetra­hydro­furan) and recrystallized from hexane. The structure has monoclinic (*P*2_1_/*n*) symmetry with a single Al atom in the asymmetric unit. The terminal C atom of one ethyl substituent is nearly equally disordered over two positions. The complex possesses catalytic activity in the ring-opening polymerization of ∊-caprolactone.

## Abstract 2

The title compound has monoclinic (*P*2_1_/*n*) symmetry with a single Al atom in the asymmetric unit. The complex possesses catalytic activity in the ring-opening polymerization of ∊-caprolactone.

## Chemical context   

Over the last decade, the number of phenoxide complexes of main group and transition metals has greatly increased due to inter­est in studies of their catalytic activity in the ring-opening polymerization (ROP) of cyclic esters (Dubois *et al.*, 2009 ▸). The design of promising new ROP catalysts bearing bulky phenoxide ligands is under way (see Sarazin & Carpentier, 2015 ▸; Nifant’ev *et al.*, 2016 ▸, 2017*b* ▸ and references therein; Chen *et al.*, 2012 ▸). One such ligand is the 4-*tert*-butyl-2,6-bis­(di­phenyl­meth­yl)phenoxide anion, [O-2,6-(Ph_2_H)_2_-4-^*t*^BuC_6_H_2_]^−^, which has recently been obtained from the corresponding phenol and characterized crystallographically as sodium salt (Searles *et al.*, 2013 ▸). However, almost all metal complexes with this ligand contain early transition metals (see below). Very recently, we have synthesized complexes with Mg, Ca, and Zn (Nifant’ev *et al.*, 2017*a* ▸), and have demonstrated their catalytic activity in the ROP of *rac*-lactide and ∊-caprolactone. Herein we report synthesis and structure of an Al complex containing this ligand.
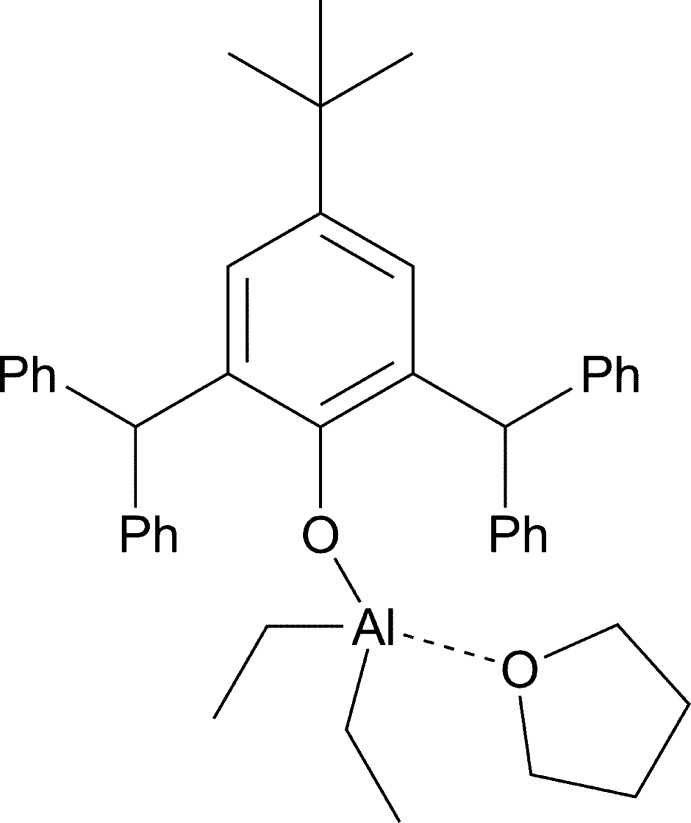


**Figure 1:**
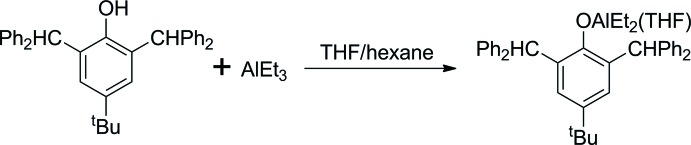
Synthesis of {Al[O-2,6-(Ph_2_CH)_2_-4-^*t*^BuC_6_H_2_]Et_2_(THF)}.

Reaction of 4-*tert*-butyl-2,6-bis­(di­phenyl­meth­yl)phenol with tri­ethyl­aluminium (1:1 molar ratio) in a hexa­ne/THF mixture followed by recrystallization from hexane leads to the formation of crystals of {Al[O-2,6-(Ph_2_CH)_2_-4-^*t*^BuC_6_H_2_]Et_2_(THF)} in 87% yield (Fig. 1 ▸).

**Figure 2:**
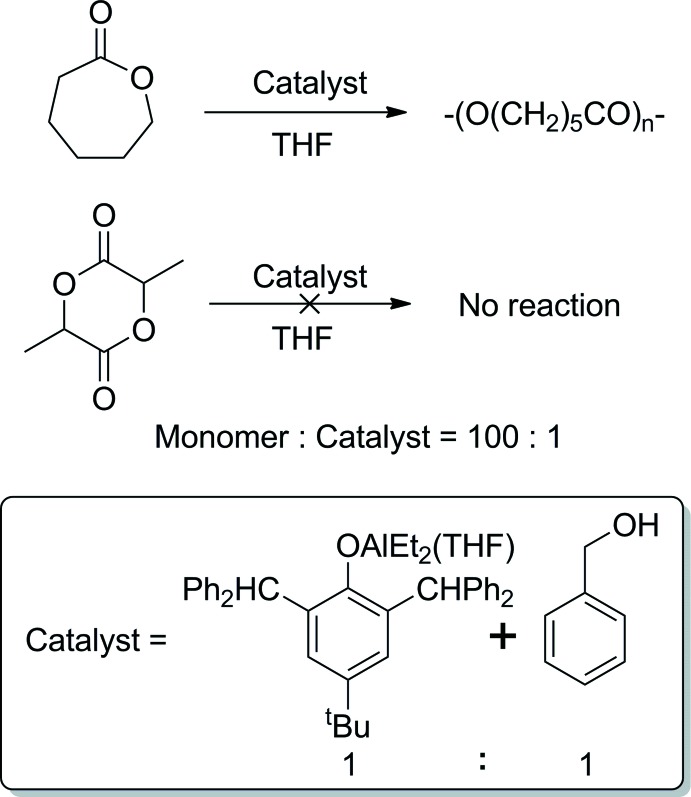
Polymerization reaction of *rac*-lactide and ∊-caprolactone.

The obtained Al complex activated by benzyl alcohol demonstrates moderate catalytic activity in *∊-*caprolactone polymerization in THF, with 14% conversion after 10 min and 100% after 4 h for a 1 *M* monomer solution (Fig. 2 ▸). However, we have found that this catalytic system is not able to catalyse the ROP of *rac*-lactide under the same conditions.

## Structural commentary   

**Figure 3:**
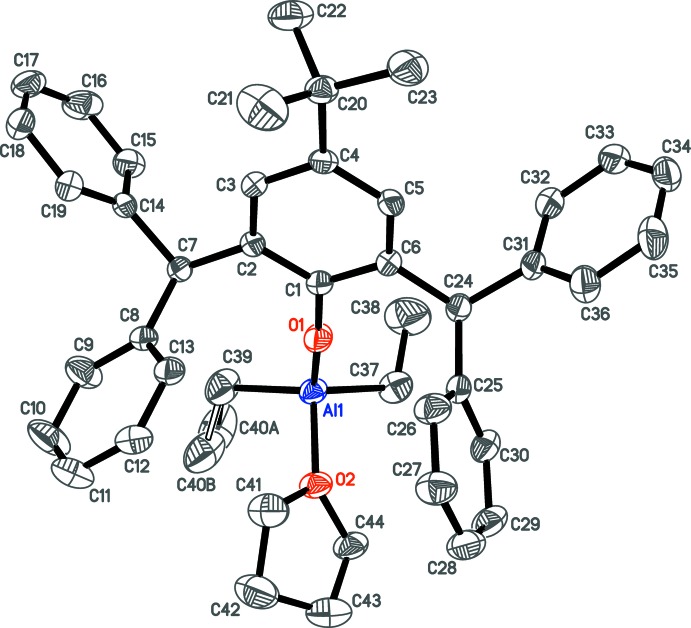
Mol­ecular structure of {Al[O-2,6-(Ph_2_CH)_2_-4-^*t*^BuC_6_H_2_]Et_2_(THF)} (50% atomic displacement ellipsoids). Hydrogen atoms are omitted for clarity. The second disorder component for one of the ethyl groups (atom C40*B*) is shown with an open solid line.

The Al atom of the title compound, {Al[O-2,6-(Ph_2_CH)_2_-4-^t^BuC_6_H_2_]Et_2_(THF)}, is in a distorted tetra­hedral environment (Fig. 3 ▸). The C40 atom of one ethyl group is equally disordered over two positions with an occupancy ratio of 0.50 (2):0.50 (2). As expected, the largest Al–ligand distances correspond to Al—Et bonds [1.9732 (19) for Al—C37 and 1.970 (2) Å for Al—C39]. The shortest Al–ligand length is for the Al—O1 bond [1.7171 (12) Å], presumably because of the presence of a negative charge at the phenoxide anion OAr^−^ regardless of its bulkiness, whereas the Al—O_THF_ bond is somewhat longer [1.8966 (13) Å, Al—O2]. The bond angles around the Al atom range from 100.55 (6)° for O1—Al1—O2 to 116.75 (10)° for C37—Al1—C39, with the O—Al—C angles lying in the middle of this range. All phenyl groups are directed away from the Al atom because of the substantial steric hindrance of the phenoxide ligand. No non-coordinating solvent mol­ecules are present in the crystal structure, and no significant non-valence inter­molecular inter­actions have been found.

## Database survey   

The crystal structures of the phenol HO-2,6-(Ph_2_CH)_2_-4-^*t*^BuC_6_H_2_ (CSD refcode BIPXEF) and of its sodium salt [NaO-2,6-(Ph_2_CH)_2_-4-^*t*^BuC_6_H_2_]_2_ (BIPXUV) have been recently established by Searles *et al.* (2013 ▸). Coordination metal complexes with the [O-2,6-(Ph_2_CH)_2_-4-^*t*^BuC_6_H_2_] anion are still poorly studied with the exception of complexes with early transition metals. Thus, according to the Cambridge Structural Database (CSD version 5.38 with updates; Groom *et al.*, 2016 ▸), 24 complexes with only *M* = Ti, V, Cr, Nb, and Ta have been reported to date: ISEWIO, RUYHEA01, UWEDEH, BIPXIJ, BIPXOP, BIPYAC, DIZNEH, DIZNIL, DIZNOR, DIZNUX, EPUJIK, QOSDEJ, QOSPEV, QOSPIZ, QOSPOF, QOSPUL, RUYHIE, RUYHOK, RUYHUQ, SONTUM, SONVAU, SONVEY, WUWHON, WUWQOW (see also Searles *et al.*, 2013 ▸, 2014*a* ▸,*b* ▸, 2015*a* ▸,*b* ▸, 2016 ▸; Solowey *et al.*, 2016 ▸). [Zn(Et)(μ-O-2,6-(Ph_2_CH)_2_-4-^*t*^BuC_6_H_2_)]_2_, [Mg(O-2,6-(Ph_2_CH)_2_-4-^*t*^BuC_6_H_2_)_2_(THF)_2_] [Ca(O-2,6-(Ph_2_CH)_2_-4-^t^BuC_6_H_2_)_2_(THF)_3_]_3_(THF)_8_ have been recently synthesized and studied by our group (CCDC numbers: 1511142–1511144; Nifant’ev *et al.*, 2017*a* ▸).

## Synthesis and crystallization   

All synthetic manipulations were performed under a purified argon atmosphere, using Schlenk glassware, dry-box tech­niques and absolute solvents. NMR spectra were recorded with a Bruker AVANCE 400 spectrometer at 298 K. C/H elemental analysis was performed with a Perkin–Elmer 2400 Series II elemental analyzer. Gel permeation chromatography (GPC) measurements were recorded on an Agilent PL-GPC 220 chromatograph equipped with a PLgel column (eluent: THF, 1 ml/min, 313 K), using universal calibration with a polystyrene standard.

### Synthesis of the complex   

A solution of AlEt_3_ in hexane (0.5 *M*, 2.0 ml, 1.0 mmol) was added dropwise to a stirred solution of HO-2,6-(Ph_2_CH)_2_-4-^*t*^BuC_6_H_2_ (0.483 g, 1.0 mmol) in THF (4 ml). The reaction mixture was stirred for 2 h. All solvent was then evaporated under reduced pressure. The microcrystalline residue was dissolved in a minimal amount of boiling hexane. After two weeks, crystals were obtained. The mother liquor was then deca­nted and the crystals were washed with hexane (2 x 0.5 ml) and dried under dynamic vacuum. The yield was 87% (559 mg, 0.87 mmol) of colourless crystals. Calculated for C_44_H_51_AlO_2_: C, 82.72%; H, 8.05%. Found: C, 82.51%; H, 8.10%. ^1^H NMR (400MHz, C_6_D_6_): δ 0.28 (4H, quadruplet, ^3^ *J*_HH_ = 8.1Hz AlC***H_2_***CH_3_), 0.78–0.86 (4H, *m*, C***H_2_***CH_2_O_THF_), 1.13 [9H, *s* –C(C***H_3_***)_3_], 1.38 (6H, *t*, ^3^ *J*_HH_ = 8.1Hz, –AlCH_2_C***H_3_***), 2.84–2.92 (4H, *m*, C***H_2_***O_THF_), 6.31 (2H, *s*, Ph_2_C***H***), 7.00 (4H, *t*, ^3^ *J*_HH_ = 7.3Hz, ***p-H***_Ph_), 7.10 (10H, *t*, ***m-H***_Ph_+***m-H***_OAr_), 7.29 (8H, *d*, ^3^ *J*_HH_ = 7.6Hz, ***o-H***_Ph_). ^13^C{^1^H} NMR (100MHz, C_6_D_6_): δ 0.54, 9.83, 24.69, 31.70, 34.23, 51.00, 70.19, 125.92, 126.29, 130.29, 131.76, 139.75, 146.13, 153.32 (see *Supporting information*).

### Polymerization experiments   

A solution of the Al complex (69 µmol) in THF was injected into a solution of a monomer [either *rac*-lactide (*rac*-LA) or ∊-caprolactone (∊-CL), 6.9 mmol] and PhCH_2_OH (69 µmol) in THF. The monomer concentration was 1.0 *M*. The reaction was carried out for 10 min and for 4 h. According to ^1^H NMR (in CDCl_3_), conversion of *rac*-LA was 0% in both cases. Conversion of ∊-CL was 14% after 10 min, and 100% after 4 h. In the latter case, the recorded ^1^H NMR spectrum showed the disappearance of the C***H_2_***OC=O resonance signal of ∊-CL at 4.14 ppm and the presence of the poly-∊-caprolactone (PCL) resonance signal at 3.98 ppm (C***H_2_***OC=O). The polymer solution was quenched with THF containing an excess of acetic acid. The polymer solution was precipitated from Et_2_O, filtered off, reprecipitated from a THF/Et_2_O mixture at 253 K, filtered off, and dried under vacuum. The isolated PCL had a regular ^1^H NMR spectrum for PCL. GPC data (THF, 313 K): *M_n_* = 1.73 × 10^4^ PDI = 1.67.

## Refinement   

Crystal data, data collection and structure refinement details are summarized in Table 1 ▸. The hydrogen atoms were positioned geometrically (C—H = 0.95 Å for aromatic, 0.98 Å for methyl, 0.99 Å for methyl­ene and 1.00 Å for tertiary H atoms) and refined as riding atoms with relative isotropic displacement parameters *U*_iso_(H)= 1.5*U*_eq_(C) for methyl H atoms and 1.2*U*_eq_(C) otherwise. A rotating group model was applied for methyl groups. Reflection (0 0 2) was affected by the beam stop, and was therefore omitted from the refinement. SADI and SIMU *SHELXL* (Sheldrick, 2015 ▸) restraints were applied for modelling the C40*A*/C40*B* disorder.

The five highest residual electron-density peaks are located at the *t*-Bu group and near THF atoms C42 and C43, pointing to some minor remaining disorder. Using a set of positional and bond-parameter restraints, estimated ratios for the *t*-Bu rotational disorder and for the disorder in the THF mol­ecule (atoms C42, C43) were found to be 0.939 (2):0.061 (2) and 0.904 (7):0.096 (7), respectively. However, the residual electron density was not sufficient to adequately model the mentioned disorders, which were therefore not included in the final crystallographic model.

## Supplementary Material

Crystal structure: contains datablock(s) I. DOI: 10.1107/S2056989018001172/pj2049sup1.cif

Structure factors: contains datablock(s) I. DOI: 10.1107/S2056989018001172/pj2049Isup2.hkl

Supporting information file. DOI: 10.1107/S2056989018001172/pj2049Isup3.cdx

1H and 13C{1H} NMR spectra. DOI: 10.1107/S2056989018001172/pj2049sup4.pdf

1H NMR spectrum in the JDX format. DOI: 10.1107/S2056989018001172/pj2049sup5.txt

13C{1H} NMR spectrum in the JDX format. DOI: 10.1107/S2056989018001172/pj2049sup6.txt

CCDC reference: 1817894

Additional supporting information:  crystallographic information; 3D view; checkCIF report

## Figures and Tables

**Table 1:** Experimental details

Crystal data	
Chemical formula	[Al(C_2_H_5_)_2_(C_36_H_33_O)(C_4_H_8_O)]
*M*_r_	638.82
Crystal system, space group	Monoclinic, *P*2_1_/*n*
Temperature (K)	150
*a*, *b*, *c* (Å)	9.9357 (13), 9.7571 (13), 38.999 (5)
β (°)	93.586 (2)
*V* (Å^3^)	3773.3 (8)
*Z*	4
Radiation type	Mo *K*α
μ (mm^−1^)	0.09
Crystal size (mm)	0.40 × 0.35 × 0.20

Data collection	
Diffractometer	Bruker SMART APEXII
Absorption correction	Multi-scan (*SADABS*; Sheldrick, 1997 ▸)
*T*_min_, *T*_max_	0.966, 0.983
No. of measured, independent and observed [*I* > 2σ(*I*)] reflections	38112, 9098, 7153
*R*_int_	0.033
(sin θ/λ)_max_ (Å^−1^)	0.661

Refinement	
*R*[*F* ^2^ > 2σ(*F* ^2^)], *wR*(*F* ^2^), *S*	0.052, 0.140, 1.04
No. of reflections	9098
No. of parameters	440
No. of restraints	19
H-atom treatment	H-atom parameters constrained
Δρ_max_, Δρ_min_ (e Å^−3^)	0.45, −0.24

Computer programs: *APEX2* and *SAINT* (Bruker, 2008 ▸), *SHELXS97* and *SHELXTL* (Sheldrick, 2008 ▸), *SHELXL2017* (Sheldrick, 2015 ▸)and *publCIF* (Westrip, 2010 ▸).

## supplementary crystallographic information

### Crystal data

**Table:**

[Al(C_2_H_5_)_2_(C_36_H_33_O)(C_4_H_8_O)]	*F*(000) = 1376
*M**_r_* = 638.82	*D*_x_ = 1.125 Mg m^−^^3^
Monoclinic, *P*2_1_/*n*	Mo *K*α radiation, λ = 0.71073 Å
*a* = 9.9357 (13) Å	Cell parameters from 8876 reflections
*b* = 9.7571 (13) Å	θ = 2.2–30.4°
*c* = 38.999 (5) Å	µ = 0.09 mm^−^^1^
β = 93.586 (2)°	*T* = 150 K
*V* = 3773.3 (8) Å^3^	Prism, colourless
*Z* = 4	0.40 × 0.35 × 0.20 mm

### Data collection

**Table:**

Bruker SMART APEXII diffractometer	9098 independent reflections
Radiation source: fine-focus sealed tube	7153 reflections with *I* > 2σ(*I*)
Graphite monochromator	*R*_int_ = 0.033
ω scans	θ_max_ = 28.0°, θ_min_ = 2.1°
Absorption correction: multi-scan (SADABS; Sheldrick, 1997)	*h* = −13→13
*T*_min_ = 0.966, *T*_max_ = 0.983	*k* = −12→12
38112 measured reflections	*l* = −51→51

### Refinement

**Table:**

Refinement on *F*^2^	Primary atom site location: structure-invariant direct methods
Least-squares matrix: full	Secondary atom site location: difference Fourier map
*R*[*F*^2^ > 2σ(*F*^2^)] = 0.052	Hydrogen site location: inferred from neighbouring sites
*wR*(*F*^2^) = 0.140	H-atom parameters constrained
*S* = 1.04	*w* = 1/[σ^2^(*F*_o_^2^) + (0.0624*P*)^2^ + 1.8357*P*] where *P* = (*F*_o_^2^ + 2*F*_c_^2^)/3
9098 reflections	(Δ/σ)_max_ = 0.001
440 parameters	Δρ_max_ = 0.45 e Å^−^^3^
19 restraints	Δρ_min_ = −0.24 e Å^−^^3^

### Special details

**Table:**

Geometry. All esds (except the esd in the dihedral angle between two l.s. planes) are estimated using the full covariance matrix. The cell esds are taken into account individually in the estimation of esds in distances, angles and torsion angles; correlations between esds in cell parameters are only used when they are defined by crystal symmetry. An approximate (isotropic) treatment of cell esds is used for estimating esds involving l.s. planes.
Refinement. Refinement of F^2^ against ALL reflections. The weighted R-factor wR and goodness of fit S are based on F^2^, conventional R-factors R are based on F, with F set to zero for negative F^2^. The threshold expression of F^2^ > 2sigma(F^2^) is used only for calculating R-factors(gt) etc. and is not relevant to the choice of reflections for refinement. R-factors based on F^2^ are statistically about twice as large as those based on F, and R- factors based on ALL data will be even larger.

### Fractional atomic coordinates and isotropic or equivalent isotropic displacement parameters (Å^2^)

**Table:**

	*x*	*y*	*z*	*U*_iso_*/*U*_eq_	Occ. (<1)
Al1	0.49790 (5)	0.32265 (5)	0.60880 (2)	0.02744 (12)	
O1	0.41331 (11)	0.47332 (12)	0.61488 (3)	0.0321 (3)	
C1	0.32620 (14)	0.57380 (15)	0.62056 (4)	0.0232 (3)	
C2	0.28061 (14)	0.59587 (15)	0.65362 (4)	0.0227 (3)	
C3	0.19413 (14)	0.70439 (16)	0.65873 (4)	0.0234 (3)	
H3	0.163470	0.718139	0.681066	0.028*	
C4	0.15005 (14)	0.79428 (15)	0.63262 (4)	0.0227 (3)	
C5	0.19566 (14)	0.77008 (16)	0.60014 (4)	0.0242 (3)	
H5	0.166924	0.829414	0.581799	0.029*	
C6	0.28190 (15)	0.66204 (16)	0.59356 (4)	0.0238 (3)	
C7	0.33456 (14)	0.50553 (16)	0.68328 (4)	0.0228 (3)	
H7	0.346015	0.411598	0.673627	0.027*	
C8	0.47382 (15)	0.55159 (16)	0.69782 (4)	0.0254 (3)	
C9	0.54383 (18)	0.4695 (2)	0.72202 (5)	0.0407 (4)	
H9	0.503715	0.387328	0.729566	0.049*	
C10	0.6717 (2)	0.5062 (2)	0.73529 (6)	0.0533 (6)	
H10	0.718594	0.448610	0.751636	0.064*	
C11	0.73113 (18)	0.6257 (2)	0.72491 (5)	0.0445 (5)	
H11	0.818548	0.650755	0.734052	0.053*	
C12	0.66264 (16)	0.70799 (18)	0.70125 (4)	0.0329 (4)	
H12	0.702795	0.790575	0.694003	0.040*	
C13	0.53444 (15)	0.67121 (17)	0.68777 (4)	0.0275 (3)	
H13	0.488083	0.729283	0.671433	0.033*	
C14	0.23441 (14)	0.49260 (16)	0.71110 (4)	0.0238 (3)	
C15	0.13885 (16)	0.38879 (18)	0.70910 (5)	0.0322 (4)	
H15	0.138694	0.324503	0.690798	0.039*	
C16	0.04341 (18)	0.3776 (2)	0.73348 (5)	0.0442 (5)	
H16	−0.021263	0.305824	0.731817	0.053*	
C17	0.04235 (18)	0.4705 (2)	0.76009 (5)	0.0433 (5)	
H17	−0.022829	0.462780	0.776800	0.052*	
C18	0.13626 (18)	0.5746 (2)	0.76236 (4)	0.0397 (4)	
H18	0.135123	0.639287	0.780552	0.048*	
C19	0.23270 (16)	0.58538 (19)	0.73812 (4)	0.0314 (4)	
H19	0.297862	0.656663	0.740056	0.038*	
C20	0.05529 (15)	0.91216 (16)	0.64069 (4)	0.0275 (3)	
C21	0.1230 (2)	1.0046 (2)	0.66799 (6)	0.0556 (6)	
H21A	0.202521	1.047611	0.658939	0.083*	
H21B	0.150549	0.949999	0.688324	0.083*	
H21C	0.059496	1.075772	0.674316	0.083*	
C22	−0.07402 (19)	0.8525 (2)	0.65444 (6)	0.0464 (5)	
H22A	−0.050894	0.798384	0.675147	0.070*	
H22B	−0.119069	0.793652	0.636885	0.070*	
H22C	−0.134545	0.927419	0.660056	0.070*	
C23	0.0138 (2)	0.9983 (2)	0.60899 (5)	0.0425 (4)	
H23A	0.093852	1.041607	0.600275	0.064*	
H23B	−0.050023	1.069317	0.615232	0.064*	
H23C	−0.029039	0.939222	0.591152	0.064*	
C24	0.33693 (15)	0.63812 (16)	0.55835 (4)	0.0252 (3)	
H24	0.331929	0.537242	0.553916	0.030*	
C25	0.48478 (15)	0.67832 (17)	0.55785 (4)	0.0267 (3)	
C26	0.53720 (17)	0.79190 (19)	0.57539 (5)	0.0351 (4)	
H26	0.480716	0.844599	0.589091	0.042*	
C27	0.67139 (18)	0.8297 (2)	0.57319 (5)	0.0409 (4)	
H27	0.705511	0.908388	0.585181	0.049*	
C28	0.75546 (18)	0.7532 (2)	0.55359 (5)	0.0398 (4)	
H28	0.846960	0.779297	0.551962	0.048*	
C29	0.70512 (18)	0.6391 (2)	0.53651 (5)	0.0389 (4)	
H29	0.762362	0.585713	0.523163	0.047*	
C30	0.57119 (17)	0.60144 (18)	0.53865 (4)	0.0324 (4)	
H30	0.537932	0.522033	0.526838	0.039*	
C31	0.24906 (16)	0.70749 (17)	0.52972 (4)	0.0269 (3)	
C32	0.12342 (17)	0.6520 (2)	0.52021 (4)	0.0349 (4)	
H32	0.096596	0.568918	0.530481	0.042*	
C33	0.03632 (18)	0.7159 (2)	0.49596 (4)	0.0403 (4)	
H33	−0.048865	0.676020	0.489656	0.048*	
C34	0.07328 (19)	0.8373 (2)	0.48102 (4)	0.0405 (4)	
H34	0.013006	0.882370	0.464853	0.049*	
C35	0.1983 (2)	0.8924 (2)	0.48974 (5)	0.0430 (4)	
H35	0.224843	0.975224	0.479265	0.052*	
C36	0.28629 (19)	0.82776 (19)	0.51380 (4)	0.0360 (4)	
H36	0.372675	0.866347	0.519385	0.043*	
C37	0.45654 (19)	0.2349 (2)	0.56378 (5)	0.0397 (4)	
H37A	0.478759	0.299435	0.545380	0.048*	
H37B	0.514314	0.152878	0.561953	0.048*	
C38	0.3092 (2)	0.1923 (3)	0.55819 (7)	0.0708 (7)	
H38A	0.293602	0.152920	0.535172	0.106*	
H38B	0.251276	0.272752	0.560367	0.106*	
H38C	0.287985	0.123910	0.575445	0.106*	
C39	0.5079 (2)	0.2015 (2)	0.64930 (6)	0.0489 (5)	
H39A	0.549655	0.253510	0.668998	0.059*	0.50 (2)
H39B	0.414722	0.178418	0.654891	0.059*	0.50 (2)
H39C	0.505157	0.258851	0.670186	0.059*	0.50 (2)
H39D	0.426939	0.142099	0.648245	0.059*	0.50 (2)
C40A	0.5844 (16)	0.0708 (10)	0.6461 (3)	0.076 (2)	0.50 (2)
H40A	0.603195	0.030664	0.668896	0.114*	0.50 (2)
H40B	0.669590	0.089546	0.635576	0.114*	0.50 (2)
H40C	0.530617	0.006462	0.631592	0.114*	0.50 (2)
C40B	0.6333 (10)	0.1103 (12)	0.6529 (3)	0.069 (2)	0.50 (2)
H40D	0.632455	0.057379	0.674213	0.104*	0.50 (2)
H40E	0.714369	0.167626	0.653399	0.104*	0.50 (2)
H40F	0.633353	0.047410	0.633269	0.104*	0.50 (2)
O2	0.67660 (11)	0.38879 (12)	0.60647 (3)	0.0321 (3)	
C41	0.73991 (19)	0.4833 (2)	0.63165 (5)	0.0450 (5)	
H41A	0.703611	0.470620	0.654504	0.054*	
H41B	0.726090	0.579735	0.624334	0.054*	
C42	0.8875 (2)	0.4444 (3)	0.63231 (6)	0.0577 (6)	
H42A	0.945857	0.522902	0.639446	0.069*	
H42B	0.907573	0.366618	0.648122	0.069*	
C43	0.9067 (2)	0.4046 (3)	0.59586 (6)	0.0546 (6)	
H43A	0.985176	0.342825	0.594466	0.066*	
H43B	0.920339	0.486585	0.581517	0.066*	
C44	0.77753 (17)	0.3322 (2)	0.58452 (5)	0.0376 (4)	
H44A	0.752501	0.350769	0.559987	0.045*	
H44B	0.786660	0.231944	0.587857	0.045*	

### Atomic displacement parameters (Å^2^)

**Table:**

	*U*^11^	*U*^22^	*U*^33^	*U*^12^	*U*^13^	*U*^23^
Al1	0.0264 (2)	0.0267 (2)	0.0298 (3)	0.00459 (19)	0.00663 (18)	0.00271 (19)
O1	0.0341 (6)	0.0352 (6)	0.0276 (6)	0.0143 (5)	0.0073 (5)	0.0031 (5)
C1	0.0203 (7)	0.0258 (7)	0.0237 (7)	0.0028 (6)	0.0028 (5)	0.0008 (6)
C2	0.0201 (7)	0.0268 (7)	0.0212 (7)	0.0011 (6)	0.0010 (5)	0.0037 (6)
C3	0.0209 (7)	0.0290 (8)	0.0206 (7)	0.0009 (6)	0.0041 (5)	0.0008 (6)
C4	0.0176 (6)	0.0243 (7)	0.0264 (7)	0.0012 (5)	0.0019 (5)	0.0011 (6)
C5	0.0214 (7)	0.0275 (8)	0.0234 (7)	0.0010 (6)	0.0002 (5)	0.0059 (6)
C6	0.0222 (7)	0.0284 (8)	0.0209 (7)	0.0000 (6)	0.0029 (5)	0.0018 (6)
C7	0.0219 (7)	0.0253 (7)	0.0213 (7)	0.0035 (6)	0.0023 (5)	0.0033 (6)
C8	0.0202 (7)	0.0307 (8)	0.0256 (7)	0.0052 (6)	0.0037 (6)	0.0016 (6)
C9	0.0303 (9)	0.0418 (10)	0.0488 (11)	−0.0013 (8)	−0.0066 (8)	0.0167 (8)
C10	0.0348 (10)	0.0570 (13)	0.0653 (14)	0.0020 (9)	−0.0190 (9)	0.0217 (11)
C11	0.0242 (8)	0.0505 (12)	0.0574 (12)	0.0013 (8)	−0.0077 (8)	0.0012 (10)
C12	0.0254 (8)	0.0333 (9)	0.0407 (9)	−0.0009 (7)	0.0070 (7)	−0.0034 (7)
C13	0.0250 (7)	0.0297 (8)	0.0282 (8)	0.0058 (6)	0.0041 (6)	0.0003 (6)
C14	0.0213 (7)	0.0292 (8)	0.0208 (7)	0.0050 (6)	0.0006 (5)	0.0068 (6)
C15	0.0284 (8)	0.0308 (9)	0.0372 (9)	−0.0001 (7)	0.0017 (7)	0.0063 (7)
C16	0.0285 (9)	0.0446 (11)	0.0606 (13)	−0.0032 (8)	0.0106 (8)	0.0186 (10)
C17	0.0315 (9)	0.0601 (12)	0.0398 (10)	0.0127 (9)	0.0154 (7)	0.0222 (9)
C18	0.0382 (9)	0.0570 (12)	0.0246 (8)	0.0140 (9)	0.0067 (7)	0.0049 (8)
C19	0.0291 (8)	0.0411 (9)	0.0242 (8)	0.0018 (7)	0.0025 (6)	0.0001 (7)
C20	0.0248 (7)	0.0260 (8)	0.0320 (8)	0.0045 (6)	0.0042 (6)	0.0018 (6)
C21	0.0569 (13)	0.0409 (11)	0.0674 (15)	0.0107 (10)	−0.0089 (11)	−0.0186 (10)
C22	0.0329 (9)	0.0449 (11)	0.0632 (13)	0.0118 (8)	0.0166 (9)	0.0149 (10)
C23	0.0405 (10)	0.0367 (10)	0.0509 (11)	0.0129 (8)	0.0087 (8)	0.0123 (8)
C24	0.0269 (7)	0.0279 (8)	0.0211 (7)	0.0023 (6)	0.0035 (6)	0.0018 (6)
C25	0.0274 (8)	0.0323 (8)	0.0205 (7)	0.0038 (6)	0.0031 (6)	0.0050 (6)
C26	0.0300 (8)	0.0382 (9)	0.0377 (9)	0.0027 (7)	0.0072 (7)	−0.0066 (7)
C27	0.0329 (9)	0.0429 (10)	0.0470 (11)	−0.0040 (8)	0.0040 (8)	−0.0073 (8)
C28	0.0282 (8)	0.0449 (11)	0.0472 (11)	0.0011 (8)	0.0104 (7)	0.0067 (8)
C29	0.0367 (9)	0.0418 (10)	0.0403 (10)	0.0072 (8)	0.0177 (8)	0.0029 (8)
C30	0.0371 (9)	0.0342 (9)	0.0268 (8)	0.0023 (7)	0.0092 (7)	0.0000 (7)
C31	0.0296 (8)	0.0330 (8)	0.0184 (7)	0.0037 (6)	0.0039 (6)	0.0001 (6)
C32	0.0314 (8)	0.0458 (10)	0.0277 (8)	−0.0023 (7)	0.0042 (7)	0.0057 (7)
C33	0.0278 (8)	0.0635 (13)	0.0295 (9)	0.0027 (8)	0.0012 (7)	0.0000 (8)
C34	0.0418 (10)	0.0551 (12)	0.0245 (8)	0.0176 (9)	0.0005 (7)	0.0017 (8)
C35	0.0575 (12)	0.0387 (10)	0.0325 (9)	0.0060 (9)	0.0005 (8)	0.0101 (8)
C36	0.0402 (9)	0.0368 (9)	0.0307 (8)	−0.0025 (8)	−0.0013 (7)	0.0049 (7)
C37	0.0419 (10)	0.0358 (10)	0.0422 (10)	−0.0026 (8)	0.0077 (8)	−0.0068 (8)
C38	0.0526 (14)	0.0809 (18)	0.0782 (18)	−0.0189 (13)	−0.0007 (12)	−0.0226 (15)
C39	0.0578 (12)	0.0416 (11)	0.0489 (11)	0.0129 (9)	0.0170 (10)	0.0166 (9)
C40A	0.130 (6)	0.040 (3)	0.062 (4)	0.036 (4)	0.036 (4)	0.014 (3)
C40B	0.081 (4)	0.060 (4)	0.072 (5)	0.031 (3)	0.034 (3)	0.036 (4)
O2	0.0260 (6)	0.0356 (6)	0.0354 (6)	0.0035 (5)	0.0070 (5)	−0.0070 (5)
C41	0.0382 (10)	0.0453 (11)	0.0516 (12)	−0.0022 (8)	0.0037 (8)	−0.0162 (9)
C42	0.0360 (11)	0.0707 (15)	0.0653 (15)	0.0005 (10)	−0.0055 (10)	−0.0120 (12)
C43	0.0314 (10)	0.0607 (14)	0.0728 (15)	0.0031 (9)	0.0106 (10)	−0.0023 (12)
C44	0.0302 (8)	0.0417 (10)	0.0423 (10)	0.0088 (7)	0.0125 (7)	−0.0025 (8)

### Geometric parameters (Å, º)

**Table:**

Al1—O1	1.7171 (12)	C24—H24	1.0000
Al1—O2	1.8966 (13)	C25—C26	1.387 (2)
Al1—C39	1.970 (2)	C25—C30	1.393 (2)
Al1—C37	1.9732 (19)	C26—C27	1.391 (2)
O1—C1	1.3355 (18)	C26—H26	0.9500
C1—C6	1.409 (2)	C27—C28	1.386 (3)
C1—C2	1.410 (2)	C27—H27	0.9500
C2—C3	1.386 (2)	C28—C29	1.376 (3)
C2—C7	1.525 (2)	C28—H28	0.9500
C3—C4	1.394 (2)	C29—C30	1.388 (2)
C3—H3	0.9500	C29—H29	0.9500
C4—C5	1.392 (2)	C30—H30	0.9500
C4—C20	1.532 (2)	C31—C36	1.388 (2)
C5—C6	1.392 (2)	C31—C32	1.390 (2)
C5—H5	0.9500	C32—C33	1.389 (2)
C6—C24	1.527 (2)	C32—H32	0.9500
C7—C14	1.523 (2)	C33—C34	1.379 (3)
C7—C8	1.530 (2)	C33—H33	0.9500
C7—H7	1.0000	C34—C35	1.377 (3)
C8—C13	1.381 (2)	C34—H34	0.9500
C8—C9	1.391 (2)	C35—C36	1.393 (3)
C9—C10	1.388 (3)	C35—H35	0.9500
C9—H9	0.9500	C36—H36	0.9500
C10—C11	1.379 (3)	C37—C38	1.524 (3)
C10—H10	0.9500	C37—H37A	0.9900
C11—C12	1.371 (3)	C37—H37B	0.9900
C11—H11	0.9500	C38—H38A	0.9800
C12—C13	1.394 (2)	C38—H38B	0.9800
C12—H12	0.9500	C38—H38C	0.9800
C13—H13	0.9500	C39—C40A	1.494 (6)
C14—C15	1.387 (2)	C39—C40B	1.531 (6)
C14—C19	1.390 (2)	C39—H39A	0.9900
C15—C16	1.389 (2)	C39—H39B	0.9900
C15—H15	0.9500	C39—H39C	0.9900
C16—C17	1.378 (3)	C39—H39D	0.9900
C16—H16	0.9500	C40A—H40A	0.9800
C17—C18	1.378 (3)	C40A—H40B	0.9800
C17—H17	0.9500	C40A—H40C	0.9800
C18—C19	1.391 (2)	C40B—H40D	0.9800
C18—H18	0.9500	C40B—H40E	0.9800
C19—H19	0.9500	C40B—H40F	0.9800
C20—C21	1.520 (3)	O2—C41	1.461 (2)
C20—C23	1.530 (2)	O2—C44	1.4669 (19)
C20—C22	1.537 (2)	C41—C42	1.514 (3)
C21—H21A	0.9800	C41—H41A	0.9900
C21—H21B	0.9800	C41—H41B	0.9900
C21—H21C	0.9800	C42—C43	1.497 (3)
C22—H22A	0.9800	C42—H42A	0.9900
C22—H22B	0.9800	C42—H42B	0.9900
C22—H22C	0.9800	C43—C44	1.507 (3)
C23—H23A	0.9800	C43—H43A	0.9900
C23—H23B	0.9800	C43—H43B	0.9900
C23—H23C	0.9800	C44—H44A	0.9900
C24—C25	1.522 (2)	C44—H44B	0.9900
C24—C31	1.531 (2)		
			
O1—Al1—O2	100.55 (6)	C26—C25—C24	122.21 (14)
O1—Al1—C39	113.79 (8)	C30—C25—C24	119.72 (15)
O2—Al1—C39	104.01 (8)	C25—C26—C27	120.91 (16)
O1—Al1—C37	114.70 (7)	C25—C26—H26	119.5
O2—Al1—C37	104.40 (7)	C27—C26—H26	119.5
C39—Al1—C37	116.75 (10)	C28—C27—C26	120.27 (18)
C1—O1—Al1	168.32 (11)	C28—C27—H27	119.9
O1—C1—C6	119.98 (13)	C26—C27—H27	119.9
O1—C1—C2	120.85 (13)	C29—C28—C27	119.34 (17)
C6—C1—C2	119.14 (13)	C29—C28—H28	120.3
C3—C2—C1	119.08 (13)	C27—C28—H28	120.3
C3—C2—C7	121.62 (13)	C28—C29—C30	120.40 (16)
C1—C2—C7	119.19 (13)	C28—C29—H29	119.8
C2—C3—C4	123.02 (13)	C30—C29—H29	119.8
C2—C3—H3	118.5	C29—C30—C25	121.01 (17)
C4—C3—H3	118.5	C29—C30—H30	119.5
C5—C4—C3	116.94 (13)	C25—C30—H30	119.5
C5—C4—C20	123.63 (13)	C36—C31—C32	118.02 (15)
C3—C4—C20	119.43 (13)	C36—C31—C24	122.95 (15)
C4—C5—C6	122.36 (13)	C32—C31—C24	118.95 (15)
C4—C5—H5	118.8	C33—C32—C31	121.19 (17)
C6—C5—H5	118.8	C33—C32—H32	119.4
C5—C6—C1	119.46 (13)	C31—C32—H32	119.4
C5—C6—C24	122.71 (13)	C34—C33—C32	120.13 (17)
C1—C6—C24	117.76 (13)	C34—C33—H33	119.9
C14—C7—C2	111.97 (12)	C32—C33—H33	119.9
C14—C7—C8	112.11 (12)	C35—C34—C33	119.40 (17)
C2—C7—C8	112.23 (12)	C35—C34—H34	120.3
C14—C7—H7	106.7	C33—C34—H34	120.3
C2—C7—H7	106.7	C34—C35—C36	120.52 (18)
C8—C7—H7	106.7	C34—C35—H35	119.7
C13—C8—C9	118.09 (15)	C36—C35—H35	119.7
C13—C8—C7	122.91 (13)	C31—C36—C35	120.71 (18)
C9—C8—C7	119.00 (15)	C31—C36—H36	119.6
C10—C9—C8	120.74 (18)	C35—C36—H36	119.6
C10—C9—H9	119.6	C38—C37—Al1	112.97 (15)
C8—C9—H9	119.6	C38—C37—H37A	109.0
C11—C10—C9	120.51 (18)	Al1—C37—H37A	109.0
C11—C10—H10	119.7	C38—C37—H37B	109.0
C9—C10—H10	119.7	Al1—C37—H37B	109.0
C12—C11—C10	119.27 (17)	H37A—C37—H37B	107.8
C12—C11—H11	120.4	C37—C38—H38A	109.5
C10—C11—H11	120.4	C37—C38—H38B	109.5
C11—C12—C13	120.41 (17)	H38A—C38—H38B	109.5
C11—C12—H12	119.8	C37—C38—H38C	109.5
C13—C12—H12	119.8	H38A—C38—H38C	109.5
C8—C13—C12	120.98 (15)	H38B—C38—H38C	109.5
C8—C13—H13	119.5	C40A—C39—Al1	116.4 (4)
C12—C13—H13	119.5	C40B—C39—Al1	114.9 (4)
C15—C14—C19	118.52 (15)	C40A—C39—H39A	108.2
C15—C14—C7	119.72 (14)	Al1—C39—H39A	108.2
C19—C14—C7	121.72 (14)	C40A—C39—H39B	108.2
C14—C15—C16	120.87 (17)	Al1—C39—H39B	108.2
C14—C15—H15	119.6	H39A—C39—H39B	107.3
C16—C15—H15	119.6	C40B—C39—H39C	108.5
C17—C16—C15	120.11 (18)	Al1—C39—H39C	108.5
C17—C16—H16	119.9	C40B—C39—H39D	108.5
C15—C16—H16	119.9	Al1—C39—H39D	108.5
C16—C17—C18	119.73 (16)	H39C—C39—H39D	107.5
C16—C17—H17	120.1	C39—C40A—H40A	109.5
C18—C17—H17	120.1	C39—C40A—H40B	109.5
C17—C18—C19	120.27 (18)	H40A—C40A—H40B	109.5
C17—C18—H18	119.9	C39—C40A—H40C	109.5
C19—C18—H18	119.9	H40A—C40A—H40C	109.5
C14—C19—C18	120.49 (17)	H40B—C40A—H40C	109.5
C14—C19—H19	119.8	C39—C40B—H40D	109.5
C18—C19—H19	119.8	C39—C40B—H40E	109.5
C21—C20—C23	108.97 (16)	H40D—C40B—H40E	109.5
C21—C20—C4	109.82 (14)	C39—C40B—H40F	109.5
C23—C20—C4	112.60 (14)	H40D—C40B—H40F	109.5
C21—C20—C22	108.86 (17)	H40E—C40B—H40F	109.5
C23—C20—C22	107.52 (14)	C41—O2—C44	110.42 (13)
C4—C20—C22	108.98 (13)	C41—O2—Al1	123.22 (10)
C20—C21—H21A	109.5	C44—O2—Al1	125.17 (11)
C20—C21—H21B	109.5	O2—C41—C42	103.24 (15)
H21A—C21—H21B	109.5	O2—C41—H41A	111.1
C20—C21—H21C	109.5	C42—C41—H41A	111.1
H21A—C21—H21C	109.5	O2—C41—H41B	111.1
H21B—C21—H21C	109.5	C42—C41—H41B	111.1
C20—C22—H22A	109.5	H41A—C41—H41B	109.1
C20—C22—H22B	109.5	C43—C42—C41	103.26 (17)
H22A—C22—H22B	109.5	C43—C42—H42A	111.1
C20—C22—H22C	109.5	C41—C42—H42A	111.1
H22A—C22—H22C	109.5	C43—C42—H42B	111.1
H22B—C22—H22C	109.5	C41—C42—H42B	111.1
C20—C23—H23A	109.5	H42A—C42—H42B	109.1
C20—C23—H23B	109.5	C42—C43—C44	104.10 (17)
H23A—C23—H23B	109.5	C42—C43—H43A	110.9
C20—C23—H23C	109.5	C44—C43—H43A	110.9
H23A—C23—H23C	109.5	C42—C43—H43B	110.9
H23B—C23—H23C	109.5	C44—C43—H43B	110.9
C25—C24—C6	111.85 (13)	H43A—C43—H43B	109.0
C25—C24—C31	112.55 (13)	O2—C44—C43	104.63 (15)
C6—C24—C31	111.60 (12)	O2—C44—H44A	110.8
C25—C24—H24	106.8	C43—C44—H44A	110.8
C6—C24—H24	106.8	O2—C44—H44B	110.8
C31—C24—H24	106.8	C43—C44—H44B	110.8
C26—C25—C30	118.06 (15)	H44A—C44—H44B	108.9
			
O2—Al1—O1—C1	−172.5 (5)	C17—C18—C19—C14	0.9 (3)
C39—Al1—O1—C1	−61.9 (5)	C5—C4—C20—C21	−120.32 (18)
C37—Al1—O1—C1	76.1 (5)	C3—C4—C20—C21	60.0 (2)
Al1—O1—C1—C6	−111.1 (5)	C5—C4—C20—C23	1.3 (2)
Al1—O1—C1—C2	70.9 (6)	C3—C4—C20—C23	−178.41 (15)
O1—C1—C2—C3	177.62 (14)	C5—C4—C20—C22	120.51 (17)
C6—C1—C2—C3	−0.4 (2)	C3—C4—C20—C22	−59.20 (19)
O1—C1—C2—C7	1.5 (2)	C5—C6—C24—C25	105.73 (17)
C6—C1—C2—C7	−176.60 (13)	C1—C6—C24—C25	−71.32 (18)
C1—C2—C3—C4	−0.4 (2)	C5—C6—C24—C31	−21.4 (2)
C7—C2—C3—C4	175.68 (14)	C1—C6—C24—C31	161.58 (14)
C2—C3—C4—C5	0.8 (2)	C6—C24—C25—C26	−38.0 (2)
C2—C3—C4—C20	−179.51 (14)	C31—C24—C25—C26	88.63 (18)
C3—C4—C5—C6	−0.3 (2)	C6—C24—C25—C30	143.55 (15)
C20—C4—C5—C6	179.97 (14)	C31—C24—C25—C30	−89.87 (18)
C4—C5—C6—C1	−0.5 (2)	C30—C25—C26—C27	1.5 (3)
C4—C5—C6—C24	−177.48 (14)	C24—C25—C26—C27	−177.03 (16)
O1—C1—C6—C5	−177.22 (14)	C25—C26—C27—C28	−0.6 (3)
C2—C1—C6—C5	0.9 (2)	C26—C27—C28—C29	−0.4 (3)
O1—C1—C6—C24	−0.1 (2)	C27—C28—C29—C30	0.5 (3)
C2—C1—C6—C24	178.00 (14)	C28—C29—C30—C25	0.4 (3)
C3—C2—C7—C14	31.4 (2)	C26—C25—C30—C29	−1.4 (2)
C1—C2—C7—C14	−152.54 (14)	C24—C25—C30—C29	177.18 (15)
C3—C2—C7—C8	−95.71 (16)	C25—C24—C31—C36	−22.4 (2)
C1—C2—C7—C8	80.35 (17)	C6—C24—C31—C36	104.31 (18)
C14—C7—C8—C13	−119.55 (16)	C25—C24—C31—C32	160.81 (15)
C2—C7—C8—C13	7.5 (2)	C6—C24—C31—C32	−72.47 (19)
C14—C7—C8—C9	61.04 (19)	C36—C31—C32—C33	−1.1 (3)
C2—C7—C8—C9	−171.93 (15)	C24—C31—C32—C33	175.83 (15)
C13—C8—C9—C10	−0.8 (3)	C31—C32—C33—C34	−0.5 (3)
C7—C8—C9—C10	178.60 (18)	C32—C33—C34—C35	1.6 (3)
C8—C9—C10—C11	0.6 (3)	C33—C34—C35—C36	−1.0 (3)
C9—C10—C11—C12	−0.1 (3)	C32—C31—C36—C35	1.7 (3)
C10—C11—C12—C13	−0.1 (3)	C24—C31—C36—C35	−175.07 (16)
C9—C8—C13—C12	0.6 (2)	C34—C35—C36—C31	−0.7 (3)
C7—C8—C13—C12	−178.82 (14)	O1—Al1—O2—C41	48.96 (14)
C11—C12—C13—C8	−0.1 (3)	C39—Al1—O2—C41	−69.03 (15)
C2—C7—C14—C15	89.07 (17)	C37—Al1—O2—C41	168.09 (14)
C8—C7—C14—C15	−143.75 (14)	O1—Al1—O2—C44	−144.71 (13)
C2—C7—C14—C19	−88.66 (17)	C39—Al1—O2—C44	97.30 (14)
C8—C7—C14—C19	38.52 (19)	C37—Al1—O2—C44	−25.59 (15)
C19—C14—C15—C16	0.1 (2)	C44—O2—C41—C42	−19.2 (2)
C7—C14—C15—C16	−177.75 (15)	Al1—O2—C41—C42	148.89 (14)
C14—C15—C16—C17	0.2 (3)	O2—C41—C42—C43	34.7 (2)
C15—C16—C17—C18	0.1 (3)	C41—C42—C43—C44	−37.4 (2)
C16—C17—C18—C19	−0.6 (3)	C41—O2—C44—C43	−3.8 (2)
C15—C14—C19—C18	−0.6 (2)	Al1—O2—C44—C43	−171.65 (13)
C7—C14—C19—C18	177.17 (14)	C42—C43—C44—O2	25.7 (2)
