# Supplementary material for: [4-tert-Butyl-2,6-bis­(di­phenyl­meth­yl)phenolato-κO]dieth­yl(tetra­hydro­furan-κO)aluminium
Source: Acta Crystallogr E Crystallogr Commun. 2018 Jan 26;74(Pt 2):221–4. doi: 10.1107/S2056989018001172 (PMC5956341; doi:10.1107/S2056989018001172)
Supplement: Supplementary file 4 [file e-74-00221-sup4.pdf]

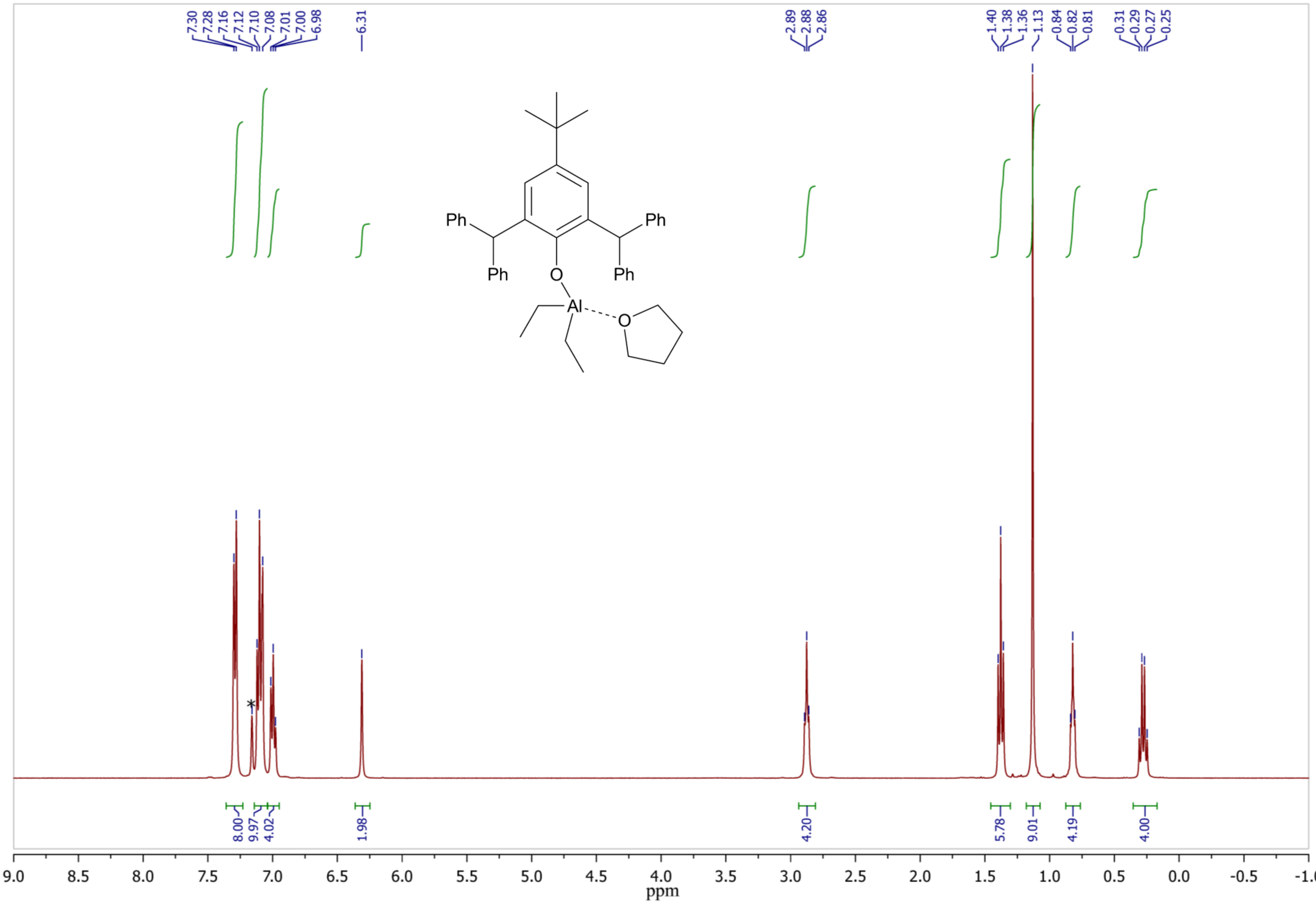

^{1}H NMR spectrum of {Al[O-2,6-(Ph$_2$CH)$_2$-4-^{t}BuC$_6$H$_2$]Et$_2$(THF)} in C$_6$D$_6$ at 400MHz.

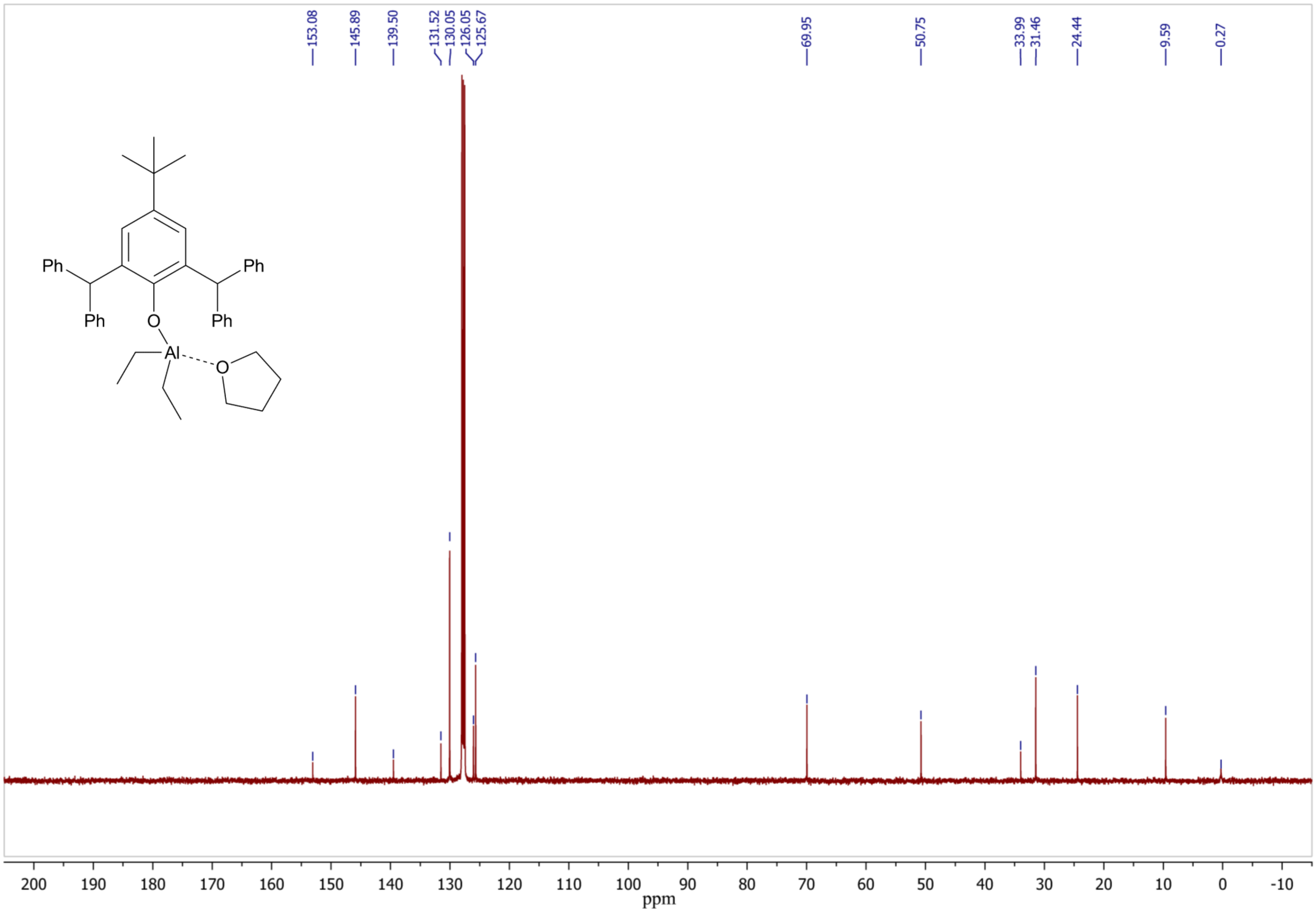

$^{13}C\{^1H\}$ NMR spectrum of $\{Al[O\text{-}2,6\text{-}(Ph_2CH)_2\text{-}4\text{-}^tBuC_6H_2]Et_2(THF)\}$ in C_6D_6 at 100MHz.
